# A High-Performance ε-Ga_2_O_3_-Based Deep-Ultraviolet Photodetector Array for Solar-Blind Imaging

**DOI:** 10.3390/ma16010295

**Published:** 2022-12-28

**Authors:** Shuren Zhou, Qiqi Zheng, Chenxi Yu, Zhiheng Huang, Lingrui Chen, Hong Zhang, Honglin Li, Yuanqiang Xiong, Chunyang Kong, Lijuan Ye, Wanjun Li

**Affiliations:** Chongqing Key Laboratory of Photo-Electric Functional Materials, College of Physics and Electronic Engineering, Chongqing Normal University, Chongqing 401331, China

**Keywords:** ε-Ga_2_O_3_, solar-blind ultraviolet photodetector, array, imaging

## Abstract

One of the most important applications of photodetectors is as sensing units in imaging systems. In practical applications, a photodetector array with high uniformity and high performance is an indispensable part of the imaging system. Herein, a photodetector array (5 × 4) consisting of 20 photodetector units, in which the photosensitive layer involves preprocessing commercial ε-Ga_2_O_3_ films with high temperature annealing, have been constructed by low-cost magnetron sputtering and mask processes. The ε-Ga_2_O_3_ ultraviolet photodetector unit shows excellent responsivity and detectivity of 6.18 A/W and 5 × 10^13^ Jones, respectively, an ultra-high light-to-dark ratio of 1.45 × 10^5^, and a fast photoresponse speed (0.14/0.09 s). At the same time, the device also shows good solar-blind characteristics and stability. Based on this, we demonstrate an ε-Ga_2_O_3_-thin-film-based solar-blind ultraviolet detector array with high uniformity and high performance for solar-blind imaging in optoelectronic integration applications.

## 1. Introduction

It is well known that deep-ultraviolet (UV) light has a wavelength of less than 280 nm. As sunlight passes through the Earth’s surface, this band of light is absorbed by a large amount of stratospheric ozone and particles, so that only a portion of the photons reach the ground through solar radiation; hence, deep-ultraviolet light is called solar-blind ultraviolet (SBUV) light [1,2,3,4]. As a result of low background noise, solar-blind ultraviolet photodetectors possess a high signal-to-noise ratio and a low false alarm rate applicable to a wide range of military and civilian applications, such as missile tracking [5], secure communications [6], and ozone hole detection [7], ultraviolet identification, fire detection [8] and high voltage discharge detection [9]. Unfortunately, the widely used photomultiplier tubes (PMTs) are limited in many fields due to their large size and high bias voltage. At present, Al_x_Ga_x−1_N [10], ZnMgO [11], diamond [12] and gallium oxide (Ga_2_O_3_) [13,14,15,16], with a bandgap greater than 4.4 eV, are considered as promising candidates for solar-blind photodetectors due to their small size, light weight and low power consumption. Among them, the percentage of aluminum (Al) element in AlGaN must exceed 40% to achieve the corresponding solar-blind zone with high wavelength requirements. However, Al element with a large specific gravity will lead to the generation of high-density defects, such as chaos, grain boundary and stacking defects, and strong parasitic reactions [5]. Similarly, the addition of Mg leads to the phase separation of ZnO, and it is difficult to achieve bands above 4.5 eV in ZnMgO with sufficient magnesium content [6,7]. The sensitivity range of diamond is limited to the narrow radiation zone below 225 nm corresponding a fixed band gap of 5.5 eV. Gallium oxide (Ga_2_O_3_) with a direct bandgap (*Eg*) of 4.9 eV, corresponding to an absorption wavelength of 254 nm, can be directly grown and prepared rather than being alloyed, which simplifies the growth of the material. Meanwhile, gallium oxide has good transparency and high thermal and chemical stability. In recent years, Ga_2_O_3_ has attracted much attention due to it being the most suitable candidate material for solar-blind photodetectors [17,18]. It is worth noting that Ga_2_O_3_ has five crystalline phases, namely α [19], β [20], ε [21], δ and γ [22] phases. Among them, ε-Ga_2_O_3_ is a metastable phase, belonging to the hexagonal crystal system and P63mc space group. It has unique advantages in the construction of photoelectric devices due to the high symmetry and low anisotropy of the crystal structure. One of the most significant applications of photodetectors (PDs) is as sensing cells in image-forming systems [23]. As is well known, PD arrays with high uniformity and device performance are essential components of high-performance imaging systems in practical applications [24].

In this study, a photodetector array consisting of 20 (5 × 4) device units was fabricated to achieve solar-blind imaging, where each sensitive unit consists of an ε-Ga_2_O_3_-based metal–semiconductor–metal (MSM) PD. It is found that the ε-Ga_2_O_3_-based SBUV PDs have high responsivity, a high light-to-dark ratio, and fast photoresponse speed and high stability. In addition, the possibility of photodetector arrays as imaging systems to obtain clear solar-blind images is explored.

## 2. Materials and Methods

### 2.1. Material Synthesis and Characterization

Commercially available ε-Ga_2_O_3_ thin films from Beijing Gallium Group were selected, which were deposited on sapphire substrates by MOCVD. The film was annealed in a single temperature zone tubular furnace under argon gas at 500 °C for 2 h. Subsequently, titanium (Ti) and gold (Au) were sequentially deposited onto the samples using a simple masking process and radio frequency magnetron sputtering. During the sputtering process, the vacuum degree was pumped to 8.0 × 10^−4^ Pa, the flow rate of argon gas was 40 sccm and the pressure in the chamber finally stabilized at 2.5 Pa. Finally, the Ti/Au electrodes were sputtered at a sputtering power of 100 W for 40 s and 3 min, respectively. Uniform MSM-type photodetector arrays (5 × 4) were constructed and a single photodetector consisted of 5 pairs of interdigital electrodes with a length of 800 μm and a spacing of 30 μm.

### 2.2. Material and Device Characterization

The crystal structure of the ε-Ga_2_O_3_ film was analyzed by an X-ray diffractometer (Bruker D8 ADVANCE A25X, Germany) using a Cu Kα line (λ = 0.1540598 nm). The absorption spectrum of the ε-Ga_2_O_3_ thin film was measured by an ultraviolet–visible–near-infrared spectrophotometer (Hitachi U-4100, Tokyo, Japan). The surface topography of the ε-Ga_2_O_3_ thin film was characterized by scanning electron microscopy (SEM). The photoelectric characteristics of the photodetectors and image-forming system tests were measured using a Keithley 2450 (America) source meter.

## 3. Results

**Figure 1:**
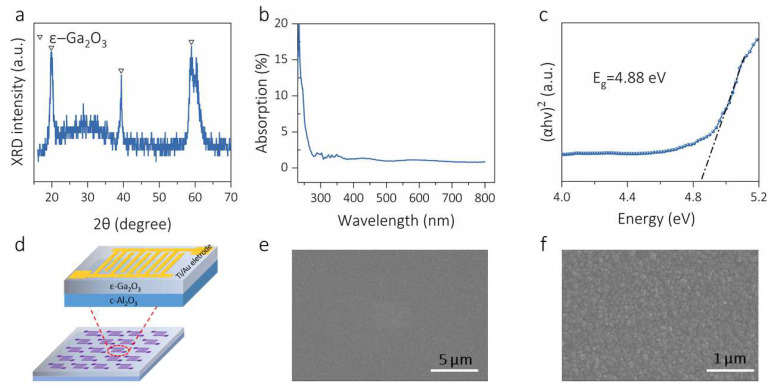
(**a**) X-ray pattern of the ԑ-Ga_2_O_3_ thin film; (**b**) absorption spectrum; (**c**) extrapolation of the bandgap diagram using the Tauc equation; (**d**) enlarged view of the photodetector array unit; (**e**,**f**) surface topography of the ε-Ga_2_O_3_ thin film.

**Figure 2:**
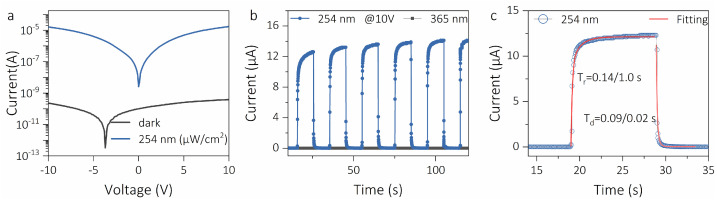
(**a**) *I*–*V* curves of the ε-Ga_2_O_3_-thin-film-based solar-blind ultraviolet photodetector under 254 nm and dark conditions. (**b**) Time-dependent photoresponse curves under 254 nm and 365 nm light. (**c**) Amplified single-period photoresponse and time fitting curve.

The ε-Ga_2_O_3_ film was annealed in an Ar atmosphere under high temperature. According to the result of the X-ray diffraction (XRD) pattern in Figure 1a, it can be seen that, except for the three diffraction peaks originating from the ε-Ga_2_O_3_ (001) direction, no other diffraction peaks are found. The three diffraction peaks located at 19.71°, 39.36° and 59.48° correspond to the (002), (004) and (006) crystal plane, respectively. This result means that the ε-Ga_2_O_3_ film annealed in an Ar atmosphere is single-phase hexagonal gallium oxide [25]. Figure 1b shows the absorption spectrum of the ε-Ga_2_O_3_ thin film. The annealed ε-Ga_2_O_3_ has an extremely low light absorption of approximately 1%, suggesting that the film has extremely high transmittance. The absorption rate below 280 nm increases sharply, showing a strong deep-ultraviolet absorption ability. In general, the optical bandgap (*Eg*) of a direct bandgap semiconductor material can be estimated from the following Tauc equation [25]:

(1) $$({\alpha h\nu )}^{2}=A\left(h\nu -E_{g}\right)$$

where the *α, hν* and *A* correspond to the absorption coefficient, the incident photon energy and a constant, respectively. The optical bandgap of the ԑ-Ga_2_O_3_ film was calculated to be 4.88 eV, as shown in Figure 1c. In order to explore the optoelectronic properties of the thin film, an MSM-type solar-blind UV photodetector array was constructed by magnetron sputtering and a simple masking technique, as shown in Figure 1d. The surface topography of the ε-Ga_2_O_3_ thin film is shown in Figure 2e,f. The film layer contains uniform grains with a size of ~100 nm and the surface is relatively flat. 

Herein, to initially evaluate the optoelectronic properties of the ε-Ga_2_O_3_ solar-blind UV detector, current–voltage *(I*–*V*) and transient photoresponse (*I*–*t*) were tested, as shown in Figure 2. Figure 2a exhibits the characteristic logarithmic *I*–*V* curves of the PD under 254 nm radiation and dark conditions. It is clear that the PD shows a super-low dark current of 350 pA at 10 V bias, which increases sharply to 18 mA under 254 nm illumination. The light-to-dark ratio of approximately 1 × 10^5^ indicates that the device has an ultra-high photoresponse to SBUV light [26]. At zero bias voltage, the dark current is 97 pA. Figure 2b displays the transient photoresponse curve of the device to periodic switching light. Under 254 nm illumination, the PD can easily switch between high-current and low-current (on/off) states, exhibiting good repeatability. By comparison, the PD is almost insensitive to periodically switched 365 nm light, indicating that the device has good photoselectivity [27]. Photoresponse time is an important performance parameter of photodetectors. Generally speaking, the photoresponse speed can be divided into rising edge (rise) and falling edge (decay), and the transient photoresponse curve can be fitted using the second-order exponential equation [28]:

(2) I=I0+Ae−tτ1+Be−tτ2

where *I*_0_ is steady state photocurrent, *A* and *B* are constants, and *τ*_1_ and *τ*_2_ are the fast and slow relaxation time constants, which corresponds to the fast generation/annihilation and capture/release processes of photogenerated carriers, respectively [5]. Figure 2c is the single-cycle magnified photoresponse image of the device. It is obvious that the photoresponse image and the second-order exponential equation have good coincidence. At the same time, the estimated rise time and fall time of the device are 0.14/1.0 s and 0.09/0.02 s, respectively, which are comparable to most of the Ga_2_O_3_-film-based solar-blind UV photodetectors reported internationally.

**Figure 3:**
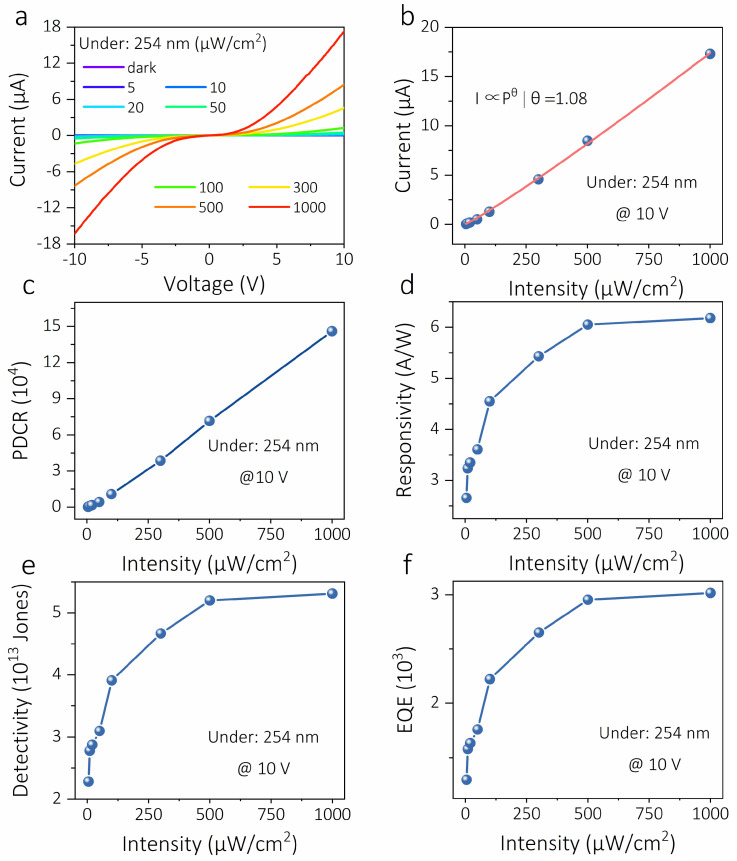
(**a**) Characteristic *I*–*V* curves of PD under 254 nm light with different light intensities. (**b**) Photocurrent, (**c**) light–dark current ratio, (**d**) responsivity, (**e**) detectivity, and (**f**) external quantum efficiency of the device as a function of light intensity under 245 nm light.

Based on *I*–*V* and *I*–*t* photoelectricity tests, it is preliminarily confirmed that the ε-Ga_2_O_3_-thin-film-based photoelectric device is a solar-blind ultraviolet photodetector with a fast photoresponse time. Subsequently, systematic optoelectronic testing and performance calculations were carried out to further evaluate the optoelectronic performance of the device, as shown in Figure 3. The photoresponse characteristics are closely related to the incident ultraviolet light intensity. Figure 3a shows the *I*–*V* characteristic curves of the PD under 254 nm illumination with different light intensities. It can be seen that the current of the PD increases monotonically with the increase in the incident light intensity under both positive and negative bias voltages, which is explained by more photogenerated electron hole pairs contributing to photocurrent being produced by a higher light intensity. Meanwhile, Ti/Au electrodes and ε-Ga_2_O_3_ thin film constitute a back-to-back Schottky contact [26,29]. In order to understand the dependence of the photocurrent and light intensity of the device in more depth, we fitted the relationship between the photocurrent and light intensity under a 10 V bias voltage according to the commonly used power-law function [30]: $I\propto P^{\theta }$, where *I* is the photocurrent of the device under 254 nm illumination (*I* = *I_light_* − *I_dark_*), *P* is the light intensity, and the index *θ* is related to the photocarrier recombination activity, as shown in Figure 3b. The *θ* for the PD is derived to be 1.08, which deviates slightly from the ideal value of 1, expressing that there is almost no recombination loss of photogenerated carriers and a small internal gain in the light intensity region [31]. In addition to the photocurrent, the photo-dark current ratio (*PDCR*) of the PD also depends on the dark current and light intensity. The *PDCR* increases with the increase in the light intensity and reaches a maximum of 1.45 × 10^5^ under 254 nm illumination with a light intensity of 1000 μW/cm^2^ (Figure 3c). In addition, the performance of PD was quantitatively calculated and compared under 254 nm illumination with different light intensities, including responsivity (*R*), detectability (D*), and external quantum efficiency (*EQE*). Thereinto, *R* is an important performance parameter reflecting the photodetector’s ability to convert light energy into electric energy. It is defined as the photocurrent generated per unit power of incident light over the effective area and is calculated by the formula [32]:

(3) $$R=\frac{I_{photo}-I_{dark}}{P_{\lambda }S}$$

where *I_photo_*, *I_dark_*, *P* and *S* correspond to the photocurrent, dark current, irradiation intensity and effective light area (2.8 × 10^−3^ cm^2^), respectively. D* is used to evaluate the device’s ability to detect weak signals in a noisy environment by relating to the noise equivalent power, which is defined as [33]:

(4) $$D=\frac{R\sqrt{S}}{\sqrt{2eI_{dark}}}$$

where *e* is the electron charge (1.6 × 10^−19^ C). *EQE* is defined as the number of electrons detected per incident photon and can be written as [34]:

(5) $$EQE=\frac{1240\times R_{254}}{\lambda }\times 100\%$$

where *λ* is the wavelength of the excitation light. Under the 254 nm illumination with a light intensity of 1000 μW/cm^2^, the *R*, D* and *EQE* of the ε-Ga_2_O_3_-thin-film-based solar-blind PD are 6.18 A/W, 5 × 10^13^ Jones and 3025, respectively. Furthermore, both *R* and *EQE* increase with light intensity, similar to those observed in others photodetector, as shown in Figure 3d–f, which again indicates that there is a small internal gain in the PD [35].

**Figure 4:**
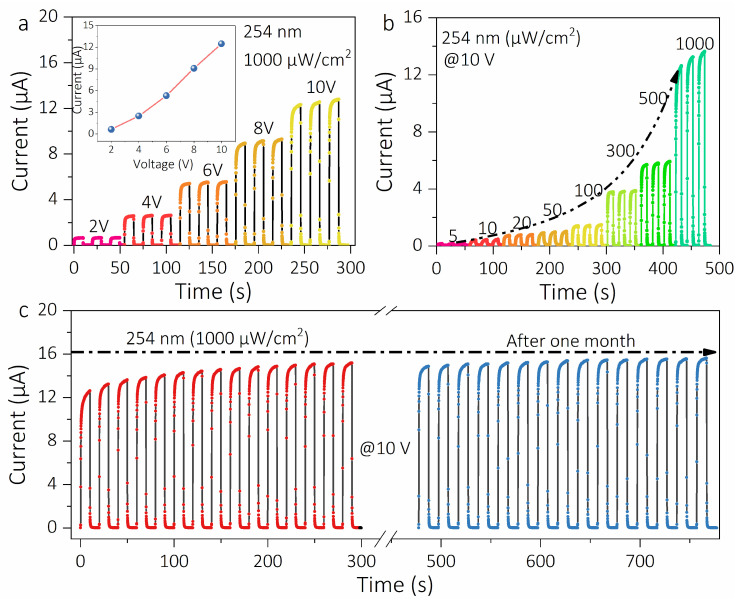
(**a**) Time-dependent curve of the ε-Ga_2_O_3_ thin-film solar-blind ultraviolet photodetector with external bias under 254 nm illumination of 1000 μW/cm^2^; (**b**) time-dependent photoresponse curves of the device under 254 nm illumination and different light intensities; (**c**) comparison diagram of the photoresponse of the device before and after one month.

Stability and repeatability are important parameters for optoelectronic devices, and the *I*–*t* curves of periodically switched deep-UV light under different bias voltages and different light intensities were further investigated. Figure 4a displays the *I*–*t* curve of the solar-blind UV photodetector under 254 nm illumination at 1000 μW/cm^2^ and different bias voltages. It is not difficult to find that the high-level current gradually increases with the increase in the applied bias voltage, and the specific change curve is shown in the inset of Figure 4a. Figure 4b depicts the *I*–*t* curve of the PD under different light intensities at 10 V, the current increases with the increase in light intensity, which is consistent with the change of photocurrent in Figure 3a. Obviously, both high external bias and light intensity can lead to an increase in the photocurrent of the device, which is due to the fact that more photogenerated electrons are generated in the photosensitive layer under both high external bias and light intensity [36]. In fact, it is very significant for industrial-grade photodetectors that they operate repeatedly for a long time and that the device remains stable. Figure 4c shows a comparison of the transient photoresponse curves of the ε-Ga_2_O_3_-film-based solar-blind UV photodetector before and after one month. It is found that the high-level current of the PD does not decay after one month, implying a good stability and reproducibility of the device. Additionally, the characteristic parameters of the previously reported MSM type solar-blind UV detector are listed in Table 1 and compared to evaluate the performance of the solar-blind UV photodetector in this work. It is not difficult to find that the device has not only a high responsivity but also a fast photoresponse speed, which is in the international medium and above level.

**Figure 5:**
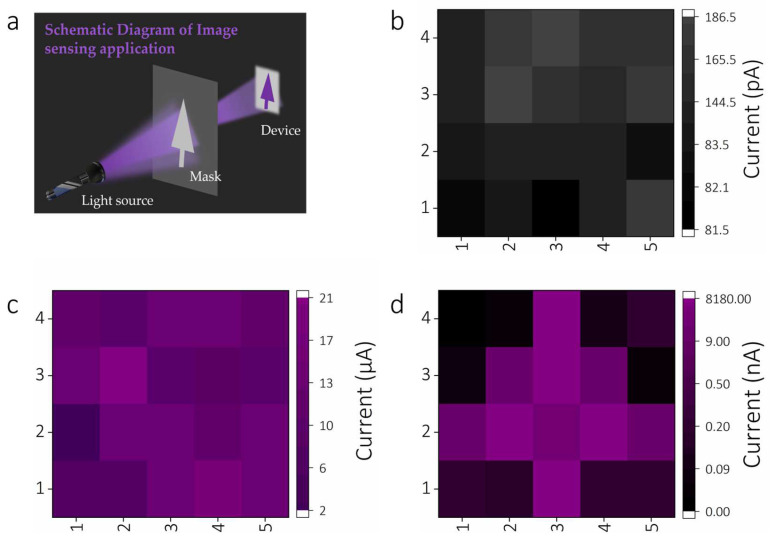
(**a**) Schematic diagram of the SBUV imaging test; 2D current contrast diagrams showing the current of the device elements of all 5 × 4 devices (**b**) in the darkness and (**c**) under 254 nm light (500 μW/cm^2^); (**d**) 2D current contrast diagram of the device array, showing the solar-blind ultraviolet imaging function.

Finally, the potential application of the PD array in solar-blind imaging is explored, and the schematic diagram of the imaging system verification principle is shown in Figure 5a. The reticle engraved with the shape of a large tree is placed in the middle of a 254 nm light source and an array of photodetectors. The ultraviolet light is irradiated on the device through the mask, and the rest is kept in the dark state or under weak ultraviolet light. The current of each PD unit is then recorded unit-by-unit by connecting the semiconductor parameter analyzer probe while keeping the test system stationary [41]. As is known, the uniformity of the photodetector array is an extremely important requirement in imaging applications. Before verifying the imaging capability, the uniformity of the optoelectronic device array (5 × 4) was obtained. The size of each pixel in the array is 1.4 mm × 1.3 mm. The photo/dark currents of each PD unit of the device array were recorded in the dark and under 254 nm light by combining the results into two-dimensional current contrast diagrams, as given in Figure 5b,c. It is clear that all PD cells can work normally with a dark current around ~0.1 nA and a photocurrent in the order of mA at 10 V. The narrow fluctuations of the dark current and photocurrent indicate the high photoresponsiveness and uniformity of the array. Subsequently, the solar-blind imaging application was verified, and the photodetector array can display a relatively clear purple tree shape (Figure 5d). The above results indicate that ε-Ga_2_O_3_-thin-film-based solar-blind photodetector arrays have the potential to be used in SBUV imaging and machine vision.

## 4. Conclusions

In conclusion, a high-performance ε-Ga_2_O_3_-thin-film-based solar-blind photodetector array with Ti/Au as the electrodes was fabricated by RF magnetron sputtering. The fabricated device exhibits excellent performance with a responsivity of 6.18 A/W, a detectivity of 5 × 10^13^ Jones, a fast photoresponse time (0.14/0.09 s), and a super-high photo-to-dark current ratio of 1.45 × 10^5^ at 10 V bias. In addition, the device shows excellent solar-blind characteristics and stability. Finally, the feasibility of the high-uniformity photodetector arrays for imaging prospects is confirmed.

## Figures and Tables

**Table 1:** Comparison of the characteristic parameters of previous MSM-type Ga_2_O_3_ film PDs.

Materials	I_dark_ (pA)	R (A/W)	τ_r_ (s)	τ_d_ (s)	Ref
β-Ga_2_O_3_	1.3 × 10^4^@10 V	/	3.39	0.6	[37]
β-Ga_2_O_3_	110@10 V	~0.001	0.31	0.05	[6]
a-Ga_2_O_3_	2.84@10 V	2.66	2.4 × 10^−5^	~0.001	[9]
a-Ga_2_O_3_	~1 × 10^6^@10 V	16.34	0.1	0.2	[38]
α/β-Ga_2_O_3_	18.5@15 V	~0.025	0.03	0.04	[39]
ε-Ga_2_O_3_	23.5@6 V	230	/	0.024	[25]
ε-Ga_2_O_3_	~0.2@5 V	1.36	0.061	0.087	[21]
ε-Ga_2_O_3_	~100@10 V	262	0.07	0.2	[40]
ε-Ga_2_O_3_	350@10 V	6.18	0.14	0.09	This work

## Data Availability

The data that support the findings of this study are available from the corresponding author upon reasonable request.
